# Validity of score interpretations on an online English placement writing test

**DOI:** 10.1186/s40468-022-00187-0

**Published:** 2022-09-15

**Authors:** Yun Deok Choi

**Affiliations:** Department of English Education, Chungnam National University, 99 Daehak-ro, Yuseong-gu, Daejeon, 34134 South Korea

**Keywords:** Computer-based testing, Computer familiarity, L2 writing ability, English placement testing, Argument-based validity

## Abstract

A much-debated question in the L2 assessment field is if computer familiarity should be considered a potential source of construct-irrelevant variance in computer-based writing (CBW) tests. This study aims to make a partial validity argument for an online source-based writing test (OSWT) designed for English placement testing (EPT), focusing on the explanation inference. Score interpretations on the OWST are proposed and supporting evidence is sought in terms of test-takers’ self-confidence in and preferences for CBW tests (two interrelated aspects of computer familiarity) and L2 writing ability. Ninety-seven ESL students demonstrating two different levels (higher and lower levels) of L2 writing ability studying at a US university completed the OSWT and an online questionnaire asking about their attitudes towards CBW tests. A series of statistical and thematic analyses revealed that most of the test-takers held self-confidence in and preferences for CBW tests for reasons related to previous CBW experience (e.g., familiarity with CBW, useful tools/functions available on computers) regardless of L2 writing ability. The higher-level test-takers obtained significantly higher scores on the OSWT than their lower-level counterparts. Test-takers’ preferences were a significant predictor of the OSWT scores only in the higher-level group. The findings largely support the validity of proposed score interpretations on the OSWT. Implications are discussed in terms of test fairness and the construct of CBW tests.

**Supplementary Information:**

The online version contains supplementary material available at 10.1186/s40468-022-00187-0.

## Introduction

Over the last few decades, the computer-based writing (CBW) test has expanded its impact on L2 assessment because of its relative advantages over paper-based writing (PBW) tests in terms of authenticity, convenience, and cost-effectiveness (Huff & Sireci, 2001). CBW has become the new norm in educational settings as computer technologies are employed extensively in writing classrooms (Hyland, 2003) and as students frequently use computers as a writing tool and complete required composition-related work on computers (e.g., Brunfaut et al., 2018; Kim et al., 2018). The increasing computer usage indicates CBW tests provide “realistic contexts for the production of student work by having the tasks and processes, as well as time and resources, parallel those in the real world” (Messick, 1994, p. 18). In addition, CBW tests are convenient, in that many test-takers can take the tests without the constraints of time and location (e.g., Kim et al., 2019). Computer testing also saves costs associated with printing and shipping paper (Way et al., 2008), although advancements in computerization and required security measures come at a high cost as well.

In this unprecedented era of COVID-19, as in-person test administrations are severely hampered, it is not a misrepresentation to state that the role of CBW tests has never been more important (Isbell & Kremmel, 2020). Some international testing companies (e.g., TOEFL iBT Home Edition) and many higher education institutions’ tests (e.g., Iowa State University’s English writing placement test) in developing and developed countries have successfully shifted assessments to online, computer-based formats in recent years (Babbar & Gupta, 2021; Isbell & Kremmel, 2020). A majority of test-takers consistently perceive this transition as positive regardless of the test-takers’ age (e.g., schoolchildren, adults) (e.g., Kim et al., 2019). The favorable attitudes seem to champion CBW testing, though further supporting evidence is needed to better justify the shift to online testing platforms.

From a traditional perspective on writing assessment, computerized testing warrants rigorous validation research because these computer-based versions might assess non-targeted constructs related to computer use experience (Chung, 2017). CBW tests involve extensive keyboard typing that can amplify undue influences of such construct-irrelevant factors, such as computer familiarity, on processes and products of the test (e.g., Chan et al., 2018; Li, 2006; McDonald, 2002; Wolfe & Manalo, 2004). Many previous studies measured different aspects of the experiential factor of CBW testing (including test-takers’ attitudes towards CBW tests) and have explored how they affect CBW test performance (e.g., Taylor et al., 1999), as there is no clear consensus on the operational definition of computer familiarity (McDonald, 2002). Findings regarding the effects of computer familiarity are mixed (e.g., Barkaoui, 2014; Karsten & Roth, 1998; Taylor et al., 1999). The observed inconsistency suggests the need for additional investigations. Moreover, McDonald (2002), who suggests that attitudes play significant roles in CBW tests as students’ willingness to use computers increases, calls for further research on the connection between attitudes and test performance, pointing out the lack of investigations and mixed findings of studies (e.g., Fulcher, 1999; Russell, 1999). Still, insufficient research inquiries have been made in response to his call. In essence, the relationship between the computer usage-related factors and CBW test performance still remains to be further explored.

In addition to the foregoing issue, there are other reasons that necessitate further study on the validity of CBW tests in relation to the aforementioned construct-irrelevant factors, particularly in English Placement Testing (EPT) contexts. Most of the prior works have examined the topic mainly at a whole-group level rather than sub-group levels, despite the possible variability in test-taker sub-groups and subsequent differences in testing results (Douglas & Hegelheimer, 2007). For instance, test-takers with advanced English as a second language (ESL) proficiency might have stronger preferences for CBW tests (Wolfe & Manalo, 2004), and such differences could positively affect CBW test scores (Lee, 2004), while less advanced ESL test-taker counterparts may have diminished preferences for and perform poorly in the new testing mode. Moreover, there are few studies that have explored the validity of computer-based testing for writing when the assessments are used to make placement decisions at English-medium colleges/universities (e.g., Kim et al., 2018; Lee, 2004). Lastly, it appears quite questionable if the traditional view that most of the literature is based on is still tenable in light of test-takers’ increasing accessibility to computers and experiences with CBW in their daily lives (e.g., Barkaoui & Knouzi, 2018; Chapelle & Douglas, 2006).

This study aims to investigate the validity of proposed score interpretations on an online source-based writing test (OSWT) that I designed for EPT from an argument-based validity perspective (e.g., Chapelle, 2021; Kane, 2013). A particular goal is to seek supporting evidence for the explanation inference by exploring if there are any differences between test-takers’ self-confidence in and preferences for CBW tests (two overlapping aspects of attitudes formed by prior computer use experience) (McDonald, 2002) and if test-takers’ preferences have a differing impact on OSWT scores depending on their levels of L2 writing ability. This research contributes to L2 writing assessment by proposing and validating interpretations of CBW test scores in EPT contexts from an evolving perspective on the construct of CBW tests.

### Literature review

#### Validity of CBW tests

One major theoretical issue that has dominated the CBW test for decades concerns its underlying construct, which has implications for score interpretations. Traditionally, educational measurement and language testing scholars claimed that writing tests should be a pure measure of writing ability regardless of testing mode (e.g., Huff & Sireci, 2001; Hunsu, 2015). From the traditional perspective, scholars have raised serious concerns regarding the validity of score-based inferences from CBW tests because test-takers have varying degrees of accessibility to computers and, resultantly, different levels of familiarity with computers can exert undue influences on CBW test performance. That is, “an assessment ought to be free of bias against all test takers, in particular by avoiding the assessment of construct-irrelevant matters” (Kunnan, 2013, p. 1105). Test-takers exposed to computers more frequently, however, are likely to yield higher test scores, which reflect composite effects of writing ability (a construct-relevant factor) and heightened computer familiarity (a construct-irrelevant factor) (e.g., Wolfe & Manalo, 2004).

By contrast, another group of scholars argues that computer familiarity should no longer be deemed a source of construct-irrelevant variance or bias in CBW tests (e.g., Chapelle & Douglas, 2006; Kim et al., 2018). Rather, they suggest that computer familiarity should be incorporated into the construct of CBW tests and that test scores be interpreted accordingly, as the ability to write using computers is integral to composition processes in contemporary school contexts (Barkaoui, 2014; Barkaoui & Knouzi, 2018). In this regard, “the idea of assessing writing ability with a paper-and-pencil writing test would be recognized by most academics as introducing bias into the measurement” (Chapelle & Douglas, 2006, p. 94). Furthermore, most students in this decade more freely access computers in and out of schools, possibly acquiring greater familiarity with CBW, without large gaps among the individuals.

Considering the ubiquity of CBW and the concomitant rich experience of writing using the computer, this study takes a more contemporary perspective and views computer familiarity as part of the construct of CBW tests.

#### Test-takers’ attitudes towards CBW tests

Test-takers’ perceptions of and preferences for computerized testing are commonly measured aspects of computer familiarity that have drawn much attention in cross-modality studies. According to the literature, an increasing number of test-taker populations at a whole-group level positively perceive or prefer CBW assessment for a range of reasons, including convenience and efficiency of the test and confidence in and adeptness at typing (e.g., Feng et al., 2019; Kim et al., 2019; Lee, 2004; Yu & Iwashita, 2021).

The two affective factors have been investigated in relation to L2 proficiency, and positive associations were widely reported in a small body of research (e.g., Brunfaut et al., 2018; Taylor et al., 1999). For instance, in a study on the computer-adaptive TOEFL, Wolfe and Manalo (2004) observed a weak but significantly positive correlation between ESL proficiency and choice of online testing mode (*r* = .25). The researchers conjectured the lower-level test-takers, who were likely to have less experience with computers and felt less comfortable with the new mode, experienced extra cognitive demands when typing on computers, a phenomenon caused by “double-translation” (p. 61): translating the L1 into English and then English to keystrokes. The additional demand could be a reason for the low-level test-takers’ preferences for PBW tests. More recently, Brunfaut et al. (2018), who explored the writing portion of the Integrated Skills in English (ISE) test suite, revealed test-takers’ general perceptions of the computer version of the test were positive across all levels of English proficiency. Interestingly, however, test-takers with more proficient English ability showed more positive perceptions of, greater expectations for, and stronger preferences for the new testing mode compared to their less proficient counterparts in English.

Taken together, the reviewed studies indicate many test-takers perceive CBW tests positively, and their perceptions of and preferences for CBW tests tend to be positively related to L2 proficiency. Extended investigations seem necessary, in that most of the research thus far has been conducted on high-stakes international tests rather than EPT.

#### Effects of computer familiarity on CBW test scores

A related area that has also been popularly explored in many questionnaire-based studies is the effect of test-takers’ computer familiarity on CBW test scores at a whole-group level; yet, the end result is inconclusive due to mixed findings. Some studies have revealed that the computer usage-related factor positively affects test scores or has interaction effects with computer use experiences on test scores (e.g., Breland et al., 2005; Lee, 2004; Yu & Iwashita, 2021). According to Jin and Yan (2017), test-takers with high levels of computer familiarity performed better on the computer version of the College English Test (CET) in China. When conducting a study on a small number of EPT test-takers who were habitual computer users, Lee (2004) discovered those who preferred CBW tests achieved higher scores on a CBW than a PBW test. On the other hand, Wolfe et al. (1996) found that while writing modes did not make a difference for test-takers with mid-to-high levels of CBW experience, those with a low level of comfort and lesser experience with computers achieved lower scores when writing on the computer than writing on paper.

In comparison, other investigations have documented that computer familiarity had no significant bearing on test scores (e.g., Fulcher, 1999; Russell, 1999). For instance, Weir et al. (2007) discovered, that overall, IELTS test-takers’ computer familiarity did not result in significant effects. Likewise, adapting Lee’s (2004) questionnaire, Choi (2021) found that test-takers’ preferences for CBW tests did not significantly contribute to scores on a CBW test that was designed to make placement decisions while graph familiarity did to a small extent. The discrepancy might have resulted from changes in the availability and use of computers in educational settings and/or the questionnaire instruments that had yet to be validated in either study.

To recap, the literature, most of which explored high-stakes international L2 testing programs, has yet to reach an agreement concerning the relationship between computer familiarity and CBW test scores. The mixed findings can be explained by various possible reasons, including different testing contexts, methodological limitations, and/or test-takers’ increasing exposure to computer usage. Further investigations are called for to gain an enhanced understanding of the relationship.

#### Argument-based validity

Argument-based validity^1^, introduced by Kane (2013), is a framework that has gained growing traction in the language testing field in the most recent decade (Chapelle & Lee, 2021), because it provides systematic guidance for conceptualizing, conducting, and interpreting the validation of language tests (Chapelle et al., 2008). Establishing argument-based validity for a test entails two consecutive steps: (1) clearly articulating intended score interpretations and uses (interpretation/use argument [I/UA]) comprised of a network of warrants, inferences, and assumptions and (2) justifying the proposed score interpretations and uses with theoretical and empirical evidence that supports the inferences listed in the I/UA (validity argument). In other words, “argument-based validation is an approach for creating a research program to investigate the validity of test score interpretation and use” (Chapelle & Lee, 2021, p.2), and an I/UA forms the basis for test validation (Chapelle et al., 2008).

**Fig. 1:**
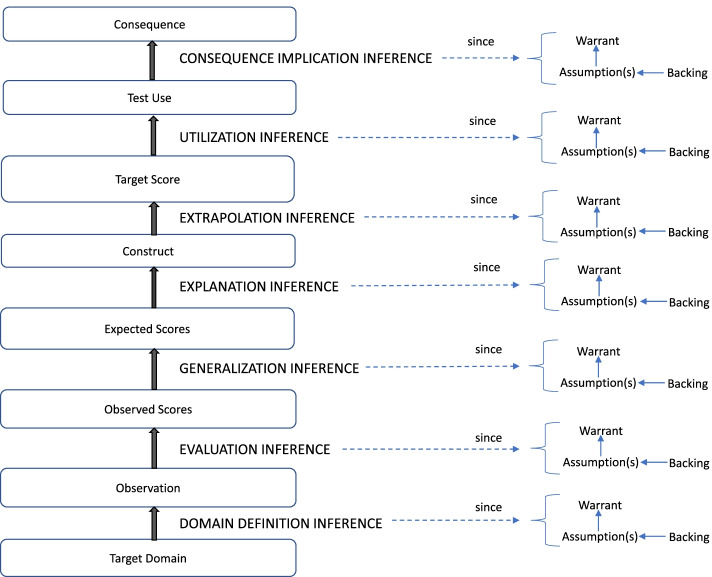
An outline of an I/UA for a language test

Figure 1 demonstrates an outline of an I/UA of a language test (Chapelle et al., 2008; Chapelle & Lee, 2021). An I/UA contains a chain of inferences that connects grounds (the target domain and observations) to intermediate conclusions/claims (the observed scores, expected scores, and construct) and, finally, to the conclusion (target score, test use, and consequence) (Chapelle et al., 2008; Chapelle & Lee, 2021). Each inference is based on a warrant (a general rule) that, in turn, depends on assumptions. We can consider a warrant a ticket that enables us to move from one inference to another (Kane, 2006). A warrant should be considered plausible when it has backing (evidence) for the assumption(s) it rests on in order to proceed from grounds to conclusions through the inferences (Chapelle et al., 2008).

#### Present study

Despite the relatively large body of research that has investigated the roles of computer familiarity in CBW test performance, our understanding of this topic seems limited and additional research is necessary, particularly in EPT contexts. Primarily, ever since the outbreak of COVID-19, a surging number of English-medium colleges and universities have migrated their in-house EPTs to online formats (Ockey, 2021). Nonetheless, the number of studies exploring the validity of CBW tests for EPT is not sufficient. Moreover, EPT test-takers’ attitudes toward CBW tests might vary depending on specific research contexts and across time due to the rapidly evolving landscape of technology use. This variability means a CBW test validated in one context might not necessarily be valid in another context, suggesting the need for ongoing context-sensitive construct validation (Fulcher, 2000). Because the placement decisions made based on online testing have substantial consequences for stakeholders, it is of importance to justify the shift to CBW tests with supporting evidence obtained from further research.

**Fig. 2:**
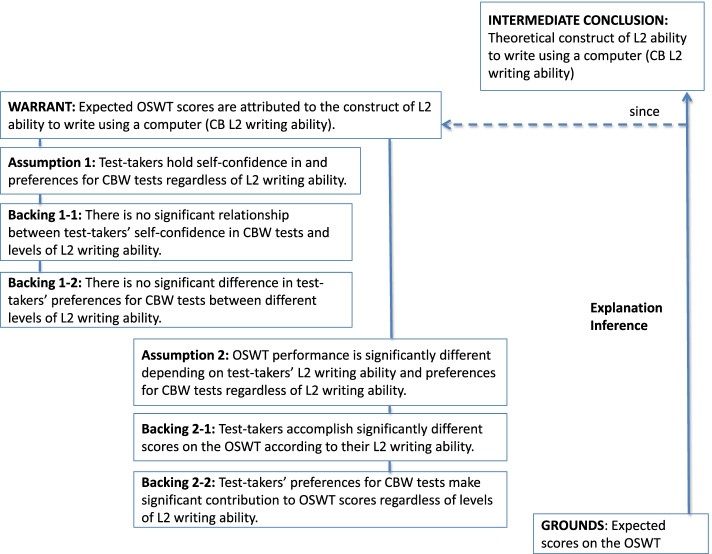
An illustration of the explanation inference for OSWT with the warrant, assumptions, and backing needed

The present research sets out to examine the validity of score interpretations on an online source-based writing test (OSWT) that I designed to make placement decisions at an English-medium university from an evolving perspective on the construct of CBW tests (e.g., Chapelle & Douglas, 2006). In the argument-based validity framework (Chapelle et al., 2008, Chapelle & Lee, 2021), this study focuses on examining the explanation inference pertinent to the construct underlying the OSWT. Figure 2 illustrates the relationships between the warrant, the assumption, and the types of backing needed to support the explanation inference.

Taking into account the ubiquity of computers and increasing CBW experience, it is assumed that most test-takers would believe that they would write better in the new testing mode rather than in the traditional, paper-based one (self-confidence in CBW tests) and hold preferences for CBW tests over PBW tests regardless of their levels of L2 writing ability (Assumption 1). This paper takes a new perspective on the construct of CBW tests (e.g., Chapelle & Douglas, 2006) — test-takers’ OSWT performance is significantly different depending on their L2 writing ability and preferences for CBW tests (Assumption 2). According to McDonald (2002), self-confidence and preferences are two related aspects of positive attitudes toward CBW tests, meaning they are not identical and possibly exert different effects on the OSWT. With the backing that links the grounds to the intermediate claim, we can claim that scores on the OSWT reflect L2 ability to write using a computer.

#### Research questions

The following research questions (RQs), derived from the assumptions of the explanation inference, guide this study.

RQ1-1: Is there a significant relationship between test-takers’ self-confidence in CBW tests and levels of L2 writing ability?

RQ1-2: Is there a significant difference between test-takers with different levels of L2 writing ability in their preferences for CBW tests?

RQ 2-1: Do test-takers with different levels of L2 writing ability perform in significantly different ways on the OSWT?

RQ 2-2: Do test-takers’ preferences for CBW tests contribute significantly to variance in their OSWT scores regardless of levels of L2 writing ability?

## Methods

The present research study used data collected as part of a larger project on the validity of an OSWT, described in detail below, to measure EPT test-takers’ computer-based L2 writing ability (Author).

### Participants

Ninety-seven ESL students taking a writing course at a large public university in the USA voluntarily participated in this study. They were newly admitted, first-year undergraduate students who were taking either a first-year composition class after passing the university’s English placement writing test or one of the two supplementary ESL writing courses (an advanced- or basic-level course) after failing the test. The university’s writing placement test assessed the writing ability to summarize two written texts and make an argument incorporating the texts and one’s experience while using academic discourse (Iowa State University, 2022). It moved from a paper-based to a CB testing mode after the COVID-19 pandemic. The other students were recruited from advanced-level writing courses at a pre-matriculated intensive English language program (IELP) at the university. The students were 34 females and 63 males of an average age of 20.05 years (*SD* = 3.62) old, a sample that was representative of the university’s EPT test-taker population in terms of L2 writing ability, L1 backgrounds (e.g., Chinese, Korean), and academic majors (e.g., Computer Science, Business, English).

Table 1 presents the number of students (*test-takers*, henceforth), as well as descriptive statistics for their TOFEL iBT writing scores, across the four writing classes. The TOEFL iBT writing section was designed to assess test-takers’ ability to synthesize written and oral sources, as well as to develop ideas, in clear discourse with good organization (Educational Testing Service, 2022). As shown in Table 1, the test-takers from the first-year composition, the advanced-level ESL, and the basic-level ESL courses achieved the highest standardized writing scores in descending order, while those from the IELP received the lowest scores.

**Table 1:** The number of test-takers and average writing scores of TOEFL iBT (*SD*s) across the four classes

	First-year composition	Advanced-level ESL	Basic-level ESL	IELP
*N*	11	36	31	19
Writing score	22.30 (3.86)	22.07 (4.51)	19.59 (3.29)	18.30 (3.66)

*Notes.* Sixteen among 97 test-takers did not submit standardized test scores (nine from the first two classes and seven from the latter two); 19 submitted IELTS writing scores and IELTS scores were converted to TOEFL iBT scores according to comparison tables (Educational Testing Service, 2010)

Due to small differences observed in the standardized writing scores between the first two and the last two courses and the small number of test-takers in each class (particularly first-year composition and IELP), I combined the former two courses into one (higher-level group, *N* = 47) and the latter two courses into another (lower-level group, *N* = 50). The two levels (higher and lower levels) were operationalized as the test-takers’ L2 writing ability. As I divided the test-takers into two groups based on the writing courses they were taking and TOEFL iBT writing scores, it was reasonable to conceive their L2 writing ability as a reflection of academic writing ability measured by the university’s writing portion of the English placement test and the TOEFL iBT writing section. Therefore, it can be said that the test-takers from the higher-level group held advanced proficiency in incorporating multiple linguistic inputs and presenting opinions about a given topic in clear, well-organized academic discourse compared to the lower-level group counterparts. An independent-samples *t*-test^2^ conducted on the TOEFL iBT writing scores indicated that there was a significant difference between the higher-level group (*M* = 21.50, *SD* = 2.68, *N* = 38) and the lower-level group (*M* = 19.11, *SD* = 3.46, *N* = 43): *t* = 3.45, *p* = .001, df = 79 with a medium to large effect size (*d* = 0.77) (Cohen, 1988,3).

Three female ESL speakers (two Korean and one Malaysian), who experienced teaching ESL courses and rating the writing section of the placement test at the university, also took part in the study as raters. Both groups of participants were provided with compensation for their participation.

### Materials

#### An online source-based writing test (OSWT)

I designed an online source-based writing test (OSWT) for EPT purposes at an English-medium university in a larger validation project (Author). The OSWT, consisting of tasks 1 and 2, provides a simple visual graph as input (Additional file 1: Appendix A). Supporting evidence for the validity of score interpretations on the OSWT as a measure of L2 writing ability was found in the earlier studies (Author). For instance, the OSWT scores were significantly associated with TOEFL iBT writing test scores when controlling for the measurement error (*r*_T1T2_ =.50), meaning both measures tapped into different aspects of the same L2 writing ability (see Author).

#### Online questionnaire

A two-part online questionnaire was crafted. The first part was designed to collect test-takers’ demographic information relevant to the study. I adapted the second part from Lee’s (2004) student survey form to capture the test-takers’ opinions about CBW tests including the OSWT (refer to Author, 2018 for details of the modification). The adapted questionnaire contained 24 items (23 close- and one open-ended). The 23 close-ended items consisted of 22 6-point Likert-scale items prompting test-takers to indicate levels of agreement with a given statement, with 1 being “strongly disagree” and 6 being “strongly agree” and one dichotomous item (item 18) that asked if test-takers expected to perform better on CBW tests over PBW tests (i.e., self-confidence in CBW tests) with two options (yes and no). Item 18 was followed by an open-ended question eliciting reasons for the presence of self-confidence in CBW tests (item 18-1). The one open-ended item (item 24) asked that test-takers provide suggestions for improvements of the OSWT (see Additional file 2: Appendix B for the adapted online questionnaire items).

After deleting three items in which the content appeared value-neutral (items 7, 8, and 15), I conducted exploratory factor analyses (EFA) on the test-takers’ responses to the 19 Likert-scale items (*α* = 0.86) prior to the main analysis to validate the questionnaire instrument. As Knekta et al. (2019) suggested, the validity of questionnaires should be established in a specific context; otherwise, valid interpretations cannot be made. The assumptions (e.g., univariate normality, absence of multicollinearity) of EFA (Ockey, 2013; Phakiti, 2018) were tenable with no missing values. Three multivariate outliers were found but were still included in the data analysis due to the fact that the unusual observations were “simply extreme cases with otherwise legitimate values” (Flora et al., 2012, p.9) and not resultant from errors related to the researcher (e.g., mistakes in data entry) or the respondents (e.g., providing the same responses across items). The sample size (*N* = 97) close to 100 was deemed acceptable (Kline, 1994).

While performing a series of EFAs with the 19 items, seven items (37%) were dropped and 12 items that tapped onto the two factors were retained, of which internal consistency was *α* = 0.94. Nine items (items 1–6, item 9, and items 16–17) measured the “test-takers’ preferences for CBW tests” factor (*α* = .95), while the other three items (items 20–22) assessed “test-takers’ perceptions of sub-writing processes of CBW tests” (*α* = .77). For the two-factor model, the Kaiser-Meyer-Olkin measure of sampling adequacy (KMO) was .92, and Bartlett’s test of sphericity was significant (*p* < .001). The identified factors explained 69.36% of the total variance.

A panel of seven experts who held advanced degrees (Ph.D. or MA) in applied linguistics reviewed the content validity of the questionnaire instrument in terms of content relevance. I calculated the content validity index (CVI) on the item and scale levels following the procedure described in Yusoff (2019). CVIs of the items and scale-level CVI were 0.86 or above, which satisfied the acceptable CVI value suggested by Lynn (1986). I used summed scores of the test-taker’ preferences for CBW tests to address RQs 1-2 and 2-2.

#### Scoring rubric

I also used a 5-point scale (1 being the least proficient and 5 being the most proficient) scoring rubric I created, pilot-tested, and validated along with the OSWT as part of the larger project (see Choi (2018) for the details of the scoring rubric development and validation). The scoring rubric contained four categories: graph description relevant to task completion, content development, organization, and use of grammar/vocabulary. The four categories were equally weighted (Additional file 3: Appendix C).

### Testing and scoring procedures

In a computer lab on campus, the test-takers signed the informed consent form and completed the counter-balanced OSWT (tasks 1 and 2); they were given 45 min per task with a 5-min intermission in between the tasks. While completing the test, the test-takers could use word processing editing functions (e.g., copy, paste) and were notified of incorrect spellings with red wavy lines underlining the text. Immediately after the test, they responded to the online questionnaire.

Raters participated in a 3-h rater training session to establish consistency in their implementation of the scoring rubric before conducting the actual ratings. During the training, the raters read over the OSWT tasks and the scoring rubric described above. Afterward, they performed practice ratings with sample essays representative of different levels of the OSWT performance. After the training, they independently judged the 194 randomly ordered test essays according to the scoring rubric. Following Shin & Ewert (2015), they completed the evaluation scale-by-scale to avoid halo effects that frequently occur in analytic ratings.

Once the raters completed ratings of the test performance, I estimated inter-rater reliability using Cronbach’s alpha, a measure of intraclass correlation (Howell, 2002). The analysis was performed in SPSS, following the procedure described in Larson-Hall (2010). According to Carr (2011), each rater-assigned score is treated as an item when Cronbach’s alpha is used to determine inter-rater agreement. Table 2 presents the inter-rater reliability for the analytic and composite scores on tasks 1 and 2. For the data analysis, I calculated composite scores for each task by averaging the scores assigned by the three raters and then averaged the two composite scores to make up the total scores for the data analyses.

**Table 2:** Inter-rater reliability for the analytic and composite scores on tasks 1 and 2

	Graph description	Content development	Organization	Use of grammar/vocabulary	Composite scores
Task 1	.93	.85	.86	.77	.90
Task 2	.93	.75	.88	.78	.90

### Analysis

The test-takers’ response scores on their self-confidence in CBW tests, preference for CBW tests, and total scores on the OSWT were analyzed descriptively and inferentially with SPSS 25 (2017). Responses to the open-ended question (item 18-1) were coded and analyzed in DeDoose (version 9.0.46).

RQ 1-1 (the relationship between the test-takers’ self-confidence in CBW tests and levels of L2 writing ability) was explored by a two-way group-independence chi-square analysis and RQ 1-2 (differences between test-takers with different levels of L2 writing ability in their preferences for CBW tests) was analyzed by an independent-samples *t*-test. RQ 2-1 (differences in test-takers’ OSWT scores depending on their levels of L2 writing ability) was addressed by performing an independent-samples *t*-test and RQ2-2 (impact of test-takers’ preferences for CBW tests on their OSWT scores) with a simple linear regression, where test-takers’ preferences for CBW tests was an independent variable and OSWT scores was a dependent variable.

Data screening conducted before the main analyses revealed that the data reasonably met the assumptions for all inferential statistical techniques: chi-square test (independence of observations, nominal data, at least five cases in each cell), independent-samples *t*-test (interval data, independent observations, normality, and equal variances), and simple linear regression (linearity, independence, normality, and constant variances) (Larson-Hall, 2010).

Furthermore, I performed a thematic analysis of responses to item 18-1 obtained from 63 test-takers who held self-confidence in CBW tests (it should be noted that one test-taker did not provide reasons) in order to uncover the similar and different reasons for their self-confidence across the two levels of L2 writing ability. The thematic analysis involved repeated data reading and coding and identification of commonly recurring themes (patterns) (Braun & Clarke, 2006). Units of analysis were words, phrases, and sentences. For the reasons for holding self-confidence, I identified five categories through iterative coding of 105 comments. Although it was not required, 12 test-takers (four who held self-confidence in CBW tests and eight who did not) provided 13 comments regarding their lack of self-confidence in CBW tests. I analyzed these comments as well and three themes emerged. The total number of comments was larger than that of the test-takers who responded to item 18-1, because some of the test-takers provided multiple reasons. A second coder double-coded 80% of the comments. Intercoder reliability estimated by a percentage of exact agreement was 86%, which is considered acceptable (Mackey & Gass, 2015). All identified disagreements were negotiated until reaching an agreement. The frequency and percentages of the categories were calculated for both levels. I used percentages to make group comparisons to address variability in the number of comments between levels.

## Results

In this section, when it comes to the inferential statistics, I will focus on effect sizes that are not affected by sample size and/or 95% CIs rather than *p*-values when applicable, in light of the relatively small number of the test-takers, following Plonsky’s (2015) suggestions. A small sample size would possibly result in insufficient power of statistical analyses to detect genuine differences in groups or relationships between variables (see Plonsky (2015) or Larson-Hall (2016) for a detailed discussion of this topic). The sample size issue holds particularly true for the simple regression analysis.

### Test-takers’ self-confidence in and preferences for CBW tests and L2 writing ability (RQs 1 and 2)

Table 3 is a contingency table that displays the frequency of choices regarding the test-takers’ self-confidence in CBW tests for the lower- and higher-level groups. Approximately, two-thirds of the test-takers held self-confidence that they would perform better in CBW tests than PBW tests at both whole- and sub-group levels. This finding suggests that a majority of test-takers tended to hold self-confidence in CBW tests regardless of levels of L2 writing ability. A two-way group-independence chi-square analysis revealed that there was no significant relationship between the two factors (*χ*^*2*^ = 0.00, df = 1, *p* = .996), with a zero effect *ω* = .00 (Cohen, 1988). This finding means the higher- and lower-level groups believe they would perform better on a CBW test to an identical extent in EPT contexts.

**Table 3:** A contingency table of the presence or absence of test-takers’ self-confidence in CBW tests across the levels

		Higher-level	Lower-level	Total
Self-confidence in CBW tests	Yes	31 (65.96%)	33 (66%)	64 (65.98%)
	No	16 (34.04%)	17 (34%)	33 (34.02%)
Total		47 (100%)	50 (100%)	97 (100%)

Descriptive statistics for the preference scores of the higher- and lower-level groups are provided in Table 4. On average, both groups tended to prefer CBW tests over PBW tests, even though the higher-level group’s preference score was slightly larger than the lower-level group. An independent-samples *t*-test showed there was no significant difference between the higher- and lower-level groups in test-takers’ preferences for CBW tests, *t* = 1.17, *p* = .24, df = 95 with a small-sized effect (*d* = 0.24) (Cohen, 1988). The effect size indicates the magnitude of the difference in preferences for CBW tests over PBW tests between the two groups was small.

**Table 4:** Descriptive statistics for test-takers’ preferences for CBW tests across the levels

	*N*	*M*	*SD*	Min–max	Skewness
Higher-level	47	36.32	11.99	11–54	−0.374
Lower-level	50	33.42	12.35	9–53	−0.357

*Notes*. The possible minimum and maximum scores were 9 and 54

Table 5 presents the categories that emerged from the thematic analysis, as well as the frequency and percent of the categories across the levels, with sample comments. Convenience in writing (e.g., ease of deletion, spell checker available on the computer), keyboard typing-related reasons (e.g., fast writing, professional or neat appearance), and familiarity with CBW were the three most prominent reasons provided by both higher- and lower-level groups. On the other hand, test-takers from both groups did not hold self-confidence in CBW tests for the primary reasons of poor typing skills and unfamiliarity with CBW.

**Table 5:** Frequency and percent of reasons for test-takers’ presence and lack of self-confidence in CBW tests and sample comments across the levels

Category		Higher-level	Lower-level	Sample comment
Positive	Benefits in composing processes	5 (9.26%)	5 (9.80%)	*I have more idea.* *It is easier organize the essay on the screen.*
	Convenience in writing	26 (48.15%)	19 (37.25%)	*It’s more easier for me to check mistakes.* *I can more easily change when I make mistakes.*
	Familiarity with CBW	6 (11.11%)	8 (15.69 %)	*Nowadays, people use computer to write essay or mail more and more often.* *It is more comfortable when I write on a computer*
	Keyboard typing-related reasons	13 (24.07%)	17 (33.33%)	*It easier to write fast.* *it is more neat as my handwriting is very messy.*
	Others	4 (7.41%)	2 (3.92%)	*Computers provide the writer with a more clean & clear user interface.* *This is what I prefer and what I think there is no certain reason*
	Subtotal	54 (100%)	51 (100%)	
Negative	Typing-related issues	3 (50%)	1 (14.29%)	*Because I am not good at typing, so actually I don't like writing on the computer.*
	Unfamiliarity with CBW	3 (50%)	5 (71.43%)	*Taking writing test has to be on papers because we learned how to write in papers.*
	Eye fatigue	0 (0%)	1 (14.29%)	*Because I am not good at typing, so actually I don't like writing on the computer.*
	Subtotal	6 (100%)	7 (100%)	

*Notes.* “Benefits for composing” includes reasons related to idea generation, organization, or longer text length; “Convenience in writing” encompasses reasons relevant to ease of deletion and useful functions available on computers for review and revising processes (e.g., spell checker); grammatical mistakes left intact in the sample comments

### Test-takers’ preferences and OSWT scores (RQs 3 and 4)

Table 6 presents the descriptive statistics for the OSWT scores in the two groups. The higher-level group achieved a slightly higher score than the lower-level group on average. When performing the independent-samples *t*-test, I found that OSWT scores of the two groups were significantly different, *t* = 2.61, *p* = .01, df = 95 with a medium-sized effect (*d* = 0.53). This finding means the test-takers with stronger L2 writing ability performed better than those with weaker L2 writing ability on the OSWT at least to some extent, suggesting that the OSWT differentiated the test-takers according to their L2 writing ability. The result provides one piece of evidence for the validity of the proposed score interpretations on the OSWT.

**Table 6:** Descriptive statistics for OSWT scores of the higher- and lower-level groups

	*N*	*M*	*SD*	Min–max	Skewness
Higher-level	47	13.58	2.03	9.67–17.50	−0.08
Lower-level	50	12.46	2.17	8.00–17.33	0.37

*Note.* The possible minimum and maximum scores were 4 and 20, respectively

A simple linear regression that I performed to investigate the contribution of test-takers’ preferences for CBW to OSWT scores indicated that in the higher-level group, 16.20% of the variance in the OSWT scores could be explained by the test-takers’ preferences for CBW tests, *F* (1, 45) = 8.725, *p* = .005 with a medium-sized effect (*r* = .40^4^) and 95% CI [0.13, 0.62]. The effect size indicates that the test-takers’ preferences for CBW tests (the predictor) could explain approximately one-sixth of the variance in the test-takers’ OSWT scores. However, it should be noted that the observed value of *r* = .40 might not be that precise, as the 95% CI is somewhat wide. For enhanced precision, the sample size should be increased (Larson-Hall, 2016). In addition, regarding the contribution of the test-takers’ preferences to their OSWT scores, the analysis yielded *t* = 2.954, *p* = .007, and BCa 95% CI [0.023, 0.119]. The identified regression equation was predicted OSWT score = 11.102 + 0.068 × (CBW test preference score). As the BCa 95% CI did not include zero and the range was quite narrow, it can be concluded that the test-takers’ preference was a significant predictor of the OSWT scores and the unstandardized regression coefficient value (*B*) was precise.

In the lower-level group, the test-takers’ preferences for CBW tests could explain almost no variance in the OSWT scores, *F* (1, 48) = 0.131, *p* = .719 with a negligible effect size (*r* = .05) and 95% CI [−0.23, 0.32]. As the 95% CI included zero but the range was quite wide, it can be concluded that the observed value might not be that accurate. A larger sample is needed to achieve improved precision (Larson-Hall, 2016). Additionally, the test-takers’ preferences were not a significant predictor of the OSWT scores, *t* = −0.362, *p* = .719 with BCa 95% CI [−0.054, 0.031]. The identified regression equation was predicted OSWT score = 12.770 – 0.009 × (CBW test preference score).

In sum, in the higher-level group, the test-takers’ preferences were a significant predictor of the OSWT scores with a medium-sized effect. By contrast, the test-takers’ preferences were not a significant predictor of the OSWT scores and the effect size was almost nil.

## Discussion

Table 7 presents a summary of the partial validity argument for the OSWT with the assumptions and backing along with the judgment of the degree of support that the backing provides for the assumptions.

**Table 7:** A summary of the partial validity argument for the OSWT with the assumptions, backing, and the judgment of the degree of support

Assumption	Backing	Degree of support
1. Test-takers hold self-confidence in CBW tests regardless of L2 writing ability.	**1-1.** A two-way group-independence chi-square analysis revealed there was no significant difference in test-takers’ self-confidence in CBW tests between the higher- and lower-level groups with a zero effect size (*χ*^*2*^ = 0.00, df = 1, *p* = .996, *ω* = .00), meaning the two groups held an identical level of self-confidence in CBW tests. The thematic analysis indicated a majority of the test-takers from the two groups held self-confidence for similar reasons (e.g., convenience in writing). A small number of the test-takers did not hold such self-confidence for almost the same reasons (e.g., unfamiliarity with CBW).	Fully supported
	**1-2.** An independent-samples *t*-test showed there was no significant difference between the higher- and lower-level groups in test-takers’ preferences for CBW tests, *t* = 1.17, *p* = .24, df = 95 with a small-sized effect (*d* = 0.24), which suggests that test-takers tended to prefer CBW tests regardless of L2 writing ability.	Fully supported
1.2. OSWT performance is significantly different depending on test-takers’ L2 writing ability and preferences for CBW tests regardless of L2 writing ability.	**2-1.** An independent-samples *t*-test showed there was a significant difference between the higher- and lower-level groups in the OSWT scores with a medium-sized effect (*t* = 2.61, *p* = .01, df = 95, *d* = 0.53), which indicates the OWST is a measure of L2 writing ability that could differentiate the test-takers according to the levels of L2 writing ability.	Fully supported
	**2-2.** A simple linear regression revealed that, in the higher-level group, test-takers’ preferences for CBW tests were a significant predictor of the OSWT scores, *t* = 2.954, *p* = .005. However, test-takers’ preferences did not significantly contribute to OSWT scores in the lower-level group, *t* = −0.362, *p* = .719. The finding means that test-takers’ preferences significantly contributed to OWST scores only in the higher-level group.	Partially supported

### Little differences in self-confidence in and preferences for CBW tests between test-takers with different L2 writing abilities (assumption 1)

As summarized in Table 7, assumption 1 underlying the explanation inference was fully supported by two pieces of evidence (backing). The present study findings suggest that, in general, EPT test-takers tended to hold self-confidence in CBW tests regardless of L2 writing ability for reasons related to prior CBW experiences (convenience in writing, familiarity with CBW, and keyboard typing). The finding lent support for results from previous research showing that the majority of test-takers held similarly positive attitudes towards CBW tests (e.g., Kim et al., 2018; Kim et al., 2019; Lee, 2004), due to useful word processing-related functions reasons, like expedited writing (e.g., insertion, deletion, and movement capacities), and the professional and neat appearance of typed texts (Brunfaut et al., 2018). The present and previous findings together indicate widespread favorable attitudes toward (self-confidence in) CBW tests among various test-taker populations (including EPT test-takers) and test-takers’ overall familiarity with CBW that involves typing and use of word processing tools.

The overall favorable attitudes towards CBW tests regardless of L2 writing ability level is not surprising in light of the ever-growing accessibility to and applications of computers for learning writing in colleges/universities (Hyland, 2003; Kim et al., 2018). Similar to other educational settings, computers are widely used in language and content courses at the university, where the test-takers were studying at the time of data collection. Due to their pervasive computer usage, most of the test-takers might have been quite familiar with CBW and had been able to acquire sufficient skill levels in typing and using basic word processing functions available on the computer. Also, when considering the fact that test-takers from both higher- and lower-level groups reported keyboard typing as one of the reasons for their positive perceptions of CBW tests, it seems that the lower-level test-takers in the present study did not suffer from cognitive overload derived from the double-translation effect (Wolfe & Manalo, 2004). The similarly positive attitudes observed across the two groups might be attributed to the lack of cognitive disadvantages the lower-level test-takers could have experienced if they had not developed typing skills.

The first two findings suggest CBW tests would not seem to introduce a serious bias to L2 writing ability measurement and can strengthen test fairness. The majority of the test-takers believed they would perform better on CBW tests and, resultantly, preferred CBW tests over PBW ones, due to their familiarity with CBW, their typing skills, and the convenient word processing functions available on the computer. Therefore, these test-takers were not likely to be disadvantaged due to the bias that results from the new testing mode. As suggested by Lee (2004), if forced to take PBW tests, they may perform poorly due to their diminished familiarity with the testing mode and not because of weak writing abilities. In this regard, what this study found not only justifies the increasing shift to CBW tests with the changing instructional environment (due to the COVID-19 pandemic) to some extent from the test-takers’ affective perspectives.

At the same time, to prevent potential measurement bias against the relatively small number of test-takers who did not hold favorable attitudes towards CBW tests because of unfamiliarity with CBW and poor typing skills, support should be extended to better justify the transitions to CBW tests. For the sake of test fairness, these test-takers should have equal opportunities to demonstrate their L2 ability to write using the computer without being hindered by the unfamiliar testing mode. To this end, it would be advisable that EPT administrators provide online tutorials before test administrations, so that the test-takers can grow accustomed to CBW by practicing typing and becoming familiar with basic word processing functions (Barkaoui, 2014; Horkay et al., 2006; Hunsu, 2015).

### Different degrees of contributions of test-takers’ preferences for CBW tests to OSWT scores depending on levels of L2 writing ability (assumption 2)

As Table 7 displays, assumption 2 is partially backed by the findings that the test-takers demonstrated significantly different performance on the OSWT, but the relationship between test-takers’ preferences for CBW tests and OSWT scores was different depending on L2 writing ability. The former provides another layer of supporting evidence for the OSWT as a measure of L2 writing ability in addition to the support reported in previous studies (Author; Author). The predictive power of the test-takers’ preferences for CBW tests observed in the higher-level group partially echoed earlier findings that test-takers with stronger computer familiarity performed better on the Internet-based CET in China (Jin & Yan, 2017) and that EPT test-takers who preferred CBW over PBW tests achieved higher scores on a CBW test (Lee, 2004).

The significant differences in OSWT scores between the higher- and lower-level groups can be explained by Pennington’s (1996) model of CBW skill development. Presumably, the test-takers from the higher-level group executed CBW skills to take advantage of the convenient functions of word processors. Resultantly, CBW became easier for the higher-level group (writing more easily). This group, in turn, practiced and produced more writing on computers (writing more), because writing became less challenging. Because of the increased practice and production, the higher-level test-takers generated higher quality writing on the computer (writing better). In comparison, as Pennington hypothesized, test-takers from the lower-level group were unable to take advantage of CBW skills like the higher-level group, probably because they were in the beginning stages of developing CBW skills. CBW skills in the lower-level test-taker group remained relatively underdeveloped, as they struggled to produce better compositions online. In fact, the higher-level group reported convenience in writing as one of the reasons for their positive perceptions of CBW tests more frequently than the lower-level group.

The last two findings of this study pertinent to the last research questions (RQs 3 and 4) in combination provide implications for the use of OSWT and its underlying construct. Firstly, OSWT should be used with caution when making placement decisions, because it tapped into different constructs depending on levels of L2 writing ability. Heterogeneous constructs mean that we can interpret OSWT scores as test-takers’ ability to “complete writing tasks on the computer” (Barkaoui, 2014, p. 243) for the higher-level group, but not for the lower-level group.

When considering the present research findings, it seems more defensible to incorporate computer familiarity into the construct of CBW tests, rather than discounting them as a source of measurement error (e.g., Chapelle & Douglas, 2006; Kim et al., 2018). The most convincing rationale is that contemporary test users aim to predict test-takers’ ability to write using computers, not in paper-and-pencil mode, because the latter is becoming more irrelevant to the academic context (Horkay et al., 2006). In addition, as Jin and Yan (2017) suggested, computer literacy is a key contextual factor that interacts with test-takers’ writing abilities in CBW tests. Consequently, the nature of the writing construct being assessed by computers is altered (Douglas & Hegelheimer, 2007). Therefore, computer familiarity, including test-takers’ attitudes, should also be defined as part of CBW tests (Kim et al., 2018), and scores on CBW tests should be interpreted accordingly.

## Conclusion

In this study, I made a partial validity argument for the OSWT designed for EPT with a focus on the explanation inference. Generally, the findings supported the assumptions of the explanation inference, upholding the validity of the proposed score interpretations on the OSWT to a large extent. The main findings were that (1) most test-takers held positive perceptions of and preferences for CBW tests regardless of L2 writing ability, (2) the test-takers achieved significantly different scores on the OSWT depending on levels of L2 writing ability, and (3) test-takers’ preferences significantly contributed to the OSWT scores only in the higher-level group. Based on the findings, I argue that shifting to CBW tests can strengthen test fairness from the test-takers’ perspectives, and the construct of CBW tests should be reconceptualized and redefined.

This study has some limitations that should be noted. Firstly, the test-takers were ESL undergraduate and pre-matriculated students; therefore, the findings might not be applicable to graduate students, another EPT test-taker population who would likely have different amounts of experience with and proficiency in CBW. Secondly, the present study relied primarily on quantitative data. More in-depth insights regarding EPT test-takers’ attitudes towards CBW tests could have been obtained by incorporating more extensive qualitative techniques like interviews. Lastly, due to the relatively small sample size, 95% CIs resulted from the simple linear regression analysis were somewhat wide, meaning the findings could have been more precise (Larson-Hall, 2016). It is advisable, thus, for future researchers to collect a larger data sample and replicate this study.

There are a few possible extensions of the present study. Above all, the current study was conducted before the COVID-19 pandemic occurred. Many English-medium colleges/universities have migrated English placement tests from PBW to CBW since the outbreak. There is less debate and more consensus on the necessity of the format shift made for the tests among test-takers. Hence, it would be interesting to conduct follow-up research and examine test-takers’ changing attitudes towards CBW tests. It is also worth examining the impact of test-takers’ computer familiarity (including attitudes towards CBW tests) on analytic scores in online source-based writing tests, because there are limited studies that have delved into the topic (e.g., Brunfaut et al., 2018). Affective factors can exert varying influences on analytic scores on online tests that involve a complex user interface (Brunfaut et al., 2018).

## Supplementary Information

Additional file 1: Appendix A. Online source-based writing test (OSWT).

Additional file 2: Appendix B. The online questionnaire items.

Additional file 3: Appendix C. The scoring rubric.

## Data Availability

The data used in this study is available from the author on reasonable request.

## Abbreviations

CBW: Computer-based writing
CET: College English Test
EFA: Exploratory factor analysis
EPT: English Placement Testing
ESL: English as a second language
IELP: Intensive English language program
ISE: Integrated Skills in English
IUA: Interpretation/use argument
KMO: Kaiser-Meyer-Olkin
OSWT: Online source-based writing test
PBW: Paper-based writing
RQ: Research question

## Footnotes

1 Refer to Chapelle (2021) for a recent discussion of the argument-based validity.

2 The data met the assumptions of the independent-samples *t*-test (interval data, independent observations, normal distribution, and equal variances) (Larson-Hall, 2010).

3 *d* = 0.2 (small), *d* = 0.5 (medium), *d* = 0.8 (large)

4 According to Cohen (1988), *r* = .10 (small), *r* = .30 (small), *r* = .50 (large).
