# Supplementary figures and images for: Validity of score interpretations on an online English placement writing test
Source: Lang Test Asia. 2022 Sep 15;12(1):42. doi: 10.1186/s40468-022-00187-0 (PMC9474279; doi:10.1186/s40468-022-00187-0)

**Appendix A**

Online source-based writing test (OSWT)

**Task 1**


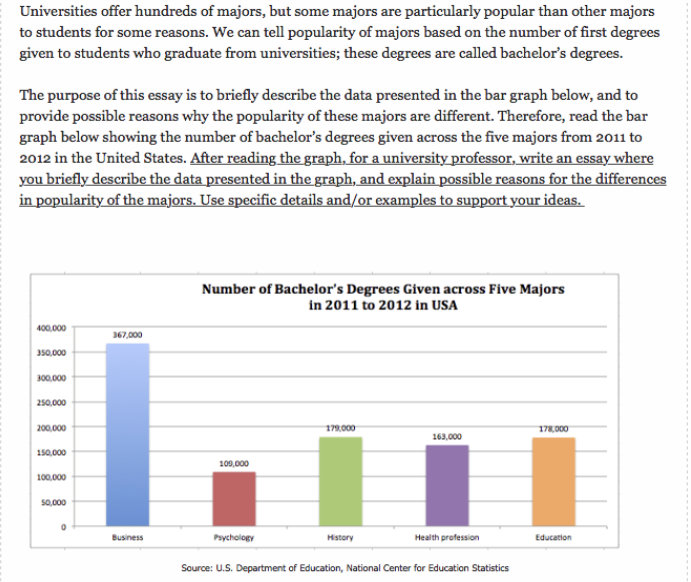


**Task 2**


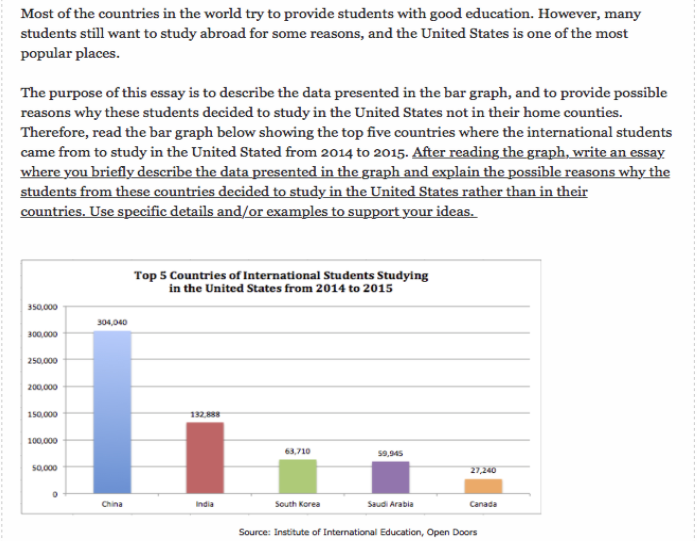

Supplement: Supplementary file 1 — Additional file 1: Appendix A. Online source-based writing test (OSWT). [file 40468_2022_187_MOESM1_ESM.docx]

**Appendix C**

The scoring rubric

**
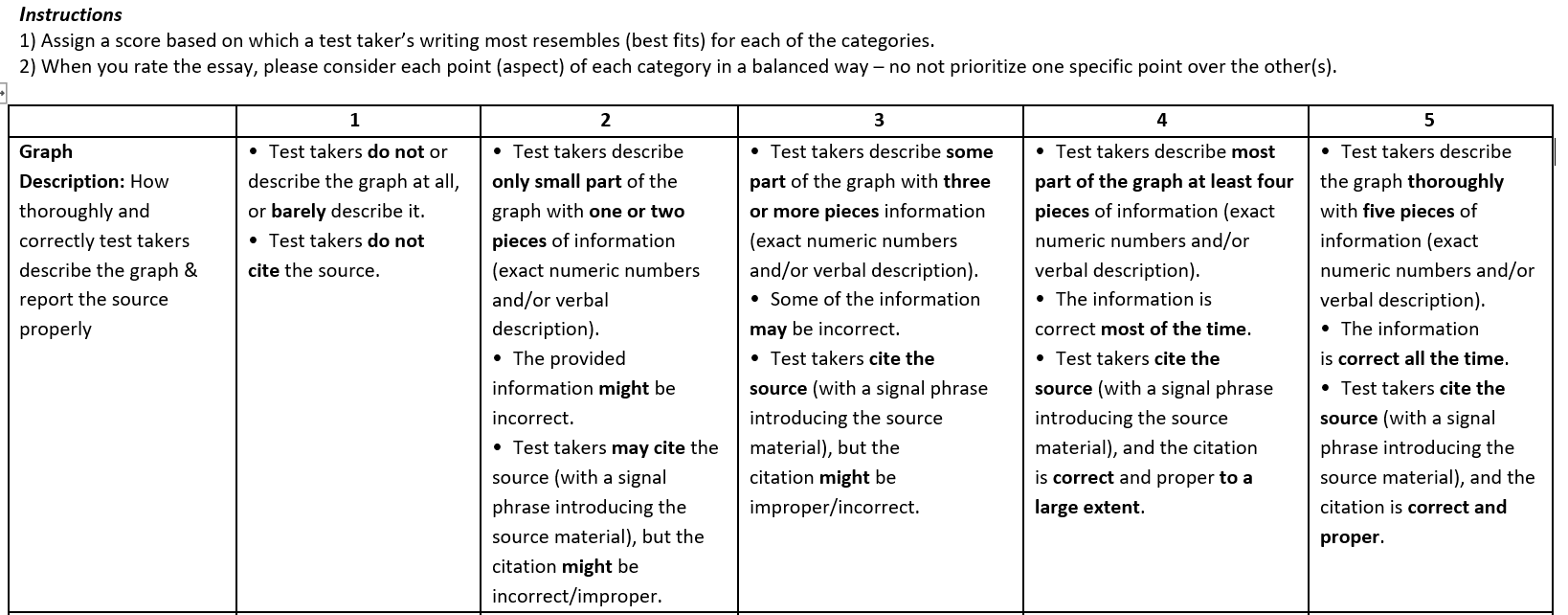
**

**
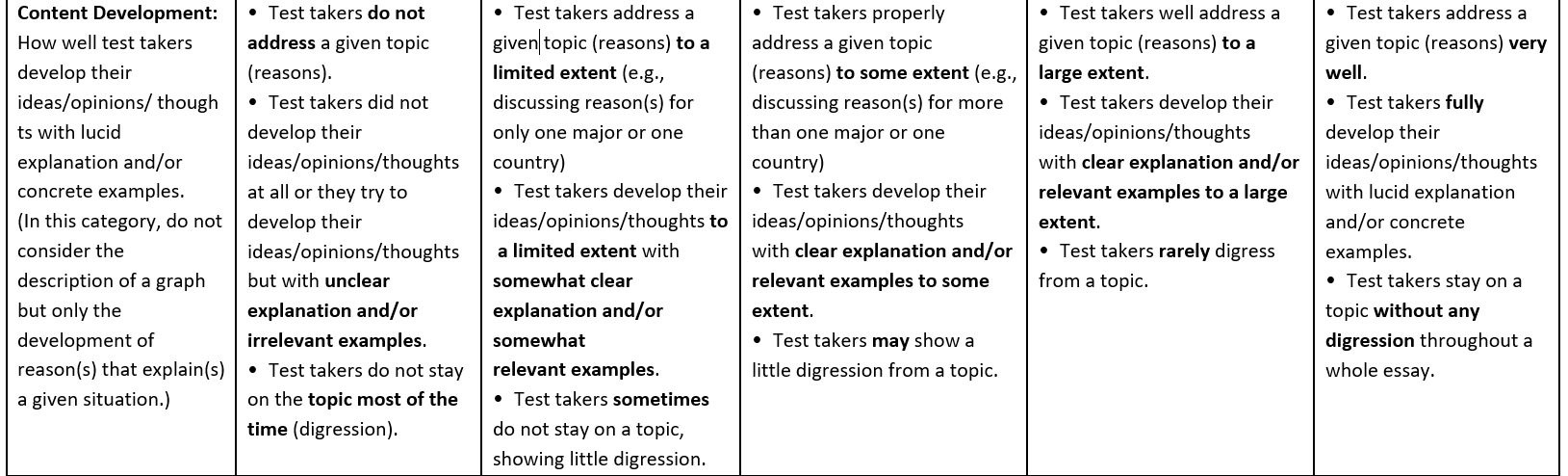
**


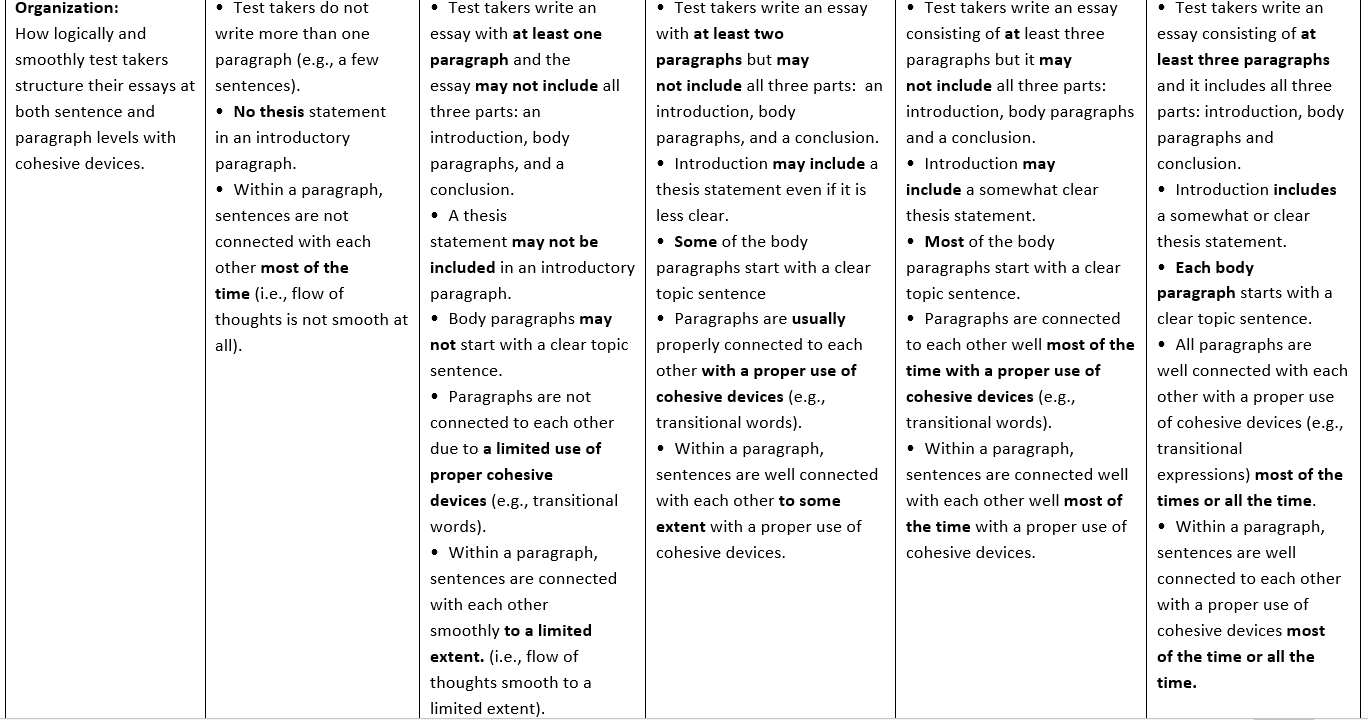


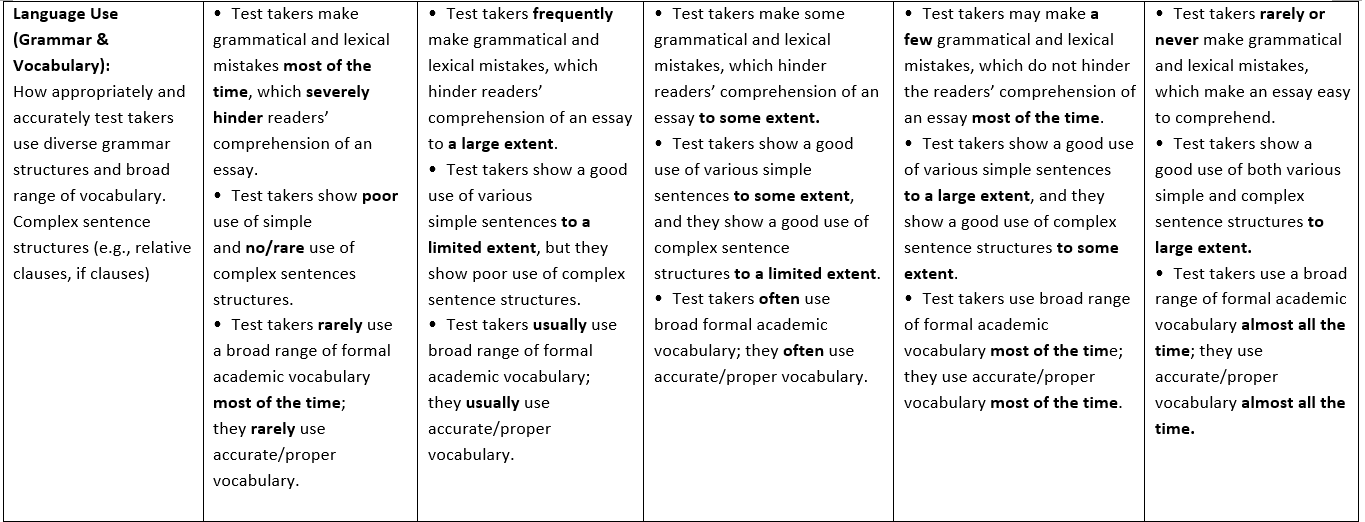

Supplement: Supplementary file 3 — Additional file 3: Appendix C. The scoring rubric. [file 40468_2022_187_MOESM3_ESM.docx]
