# Supplementary material for: Validity of score interpretations on an online English placement writing test
Source: Lang Test Asia. 2022 Sep 15;12(1):42. doi: 10.1186/s40468-022-00187-0 (PMC9474279; doi:10.1186/s40468-022-00187-0)
Supplement: Supplementary file 2 — Additional file 2: Appendix B. The online questionnaire items. [file 40468_2022_187_MOESM2_ESM.docx]

**Appendix B**

The online questionnaire items

| 1. *I prefer a computer-based writing test than a paper-based writing test. 2. *I can write better at a computer-based writing test than at a paper-based writing test. 3. *I feel more comfortable at a computer-based writing test. 4. *A computer-based writing test provides an easier writing situation than a paper-based writing test. 5. *Writing on a computer better represents my habitual writing situation than a paper-based writing test. 6. *I like a computer-based test than a paper-based test. 7. I usually use both writing methods (writing on a paper and on a computer) when I compose an essay. 8. I think my writing processes differs when I write on a computer from when I do on a paper. 9. *I usually write better when I write on a computer. 10. I expect that I will be misplaced into an ESL writing class if my score on a computer-based test is used. 11. When I compose an essay on a computer, I spend more time organizing ideas than writing on a paper. 12. I spend more time translating my ideas into texts on a computer than on a paper-based test. 13. I spend more time reviewing the texts when I compose an essay on a paper. 14. At a computer-based writing, I spend more time correcting spelling and grammatical errors than on a paper-based writing. 15. In my case, the writing tool does not make any difference in terms of the writing quality. 16. *Most of the time, I type on a computer when I compose an essay or do writing. 17. *I expect that I will be placed appropriately based on a computer test score rather than a paper-based test score. 18. Do you think you write better at the computer-delivered writing test?   18-1. Why do you feel like that? Please explain.   1. On a computer, I wrote better because I was able to write more sentences. 2. *On a computer, I wrote better because I was able to organize better. 3. *I wrote better because I was able to correct more errors. 4. *I wrote better because reviewing the text was easier. 5. I wrote better because functions in a computer such as ‘copy and paste’ prompt easier text development. 6. If you have any comments or suggestions regarding the computer-based writing test, please write.   Note. The retained 12 6-point Likert-scale items are denoted with an asterisk. |
| --- |
